# Supplementary material for: Microbe-Derived Antioxidants Reduce Lipopolysaccharide-Induced Inflammatory Responses by Activating the Nrf2 Pathway to Inhibit the ROS/NLRP3/IL-1β Signaling Pathway
Source: Int J Mol Sci. 2022 Oct 18;23(20):12477. doi: 10.3390/ijms232012477 (PMC9603940; doi:10.3390/ijms232012477)
Supplement: Supplementary file 1 [file ijms-23-12477-s001.zip › ijms-1868241-supplementary/Supplementary Tables S1 and S2.pdf]

Table S1. Primers used in current study.

Gene	Forward Primer (5'-3')	Reverse Primer (5'-3')
NLRP3	AGTGGATAGGTTTGCTGGGATA	CTGGGTGTAGCGTCTGTTGAG
ASC	CTCTGTATGGCAATGTGCTGAC	GAACAAGTTCTTGCAGGTCAG
Casepase-1	AGTGTAGGGACAATAAATGG	GATGGACCTGACTGAAGC
TNF-α	TATGGCTCAGGGTCCAACTC	GGAAAGCCCATTTGAGTCCT
IL-6	CACGGCCTTCCCTACTTCAC	TGCAAGTGCATCATCGTTGT
IL-18	GTGAACCCCAGACCAGACTG	CCTGGAACACGTTTCTGAAAGA
IL-1β	TTCTTTGAGGCTGACAGACC	CGTCTTTCATCACACAGGAC
Nrf2	AGCAGGACATGGAGCAAGTT	TTCTTTTTCCAGCGAGGAGA
NQO-1	TTCTGTGGCTTCCAGGTCTT	AGGCTGCTTGGAGCAAAATA
HO-1	CCCACCAAGTTCAAACAGCTC	AGGAAGGCGGTCTTAGCCTC
β-actin	CGGTTCCGATGCCCTGAGGCTCTT	CGTCACACTTCATGATGGAATTGA

Table S2. siRNAs fragment sequence used in current study.

Gene	Forward Primer (5'-3')	Reverse Primer (5'-3')
si NLRP3	CGGCCUUACUUCAAUCUGUTT	ACAGAUUGAAGUAAGGCCGTT
si Nrf2	GCAACUGUGGUCCACAUUUTT	AAAUGUGGACCACAGUUGCTT
